# Combined MIRACLE2 and CAHP Scores Enhance Mortality Prediction in Out-of-Hospital Cardiac Arrest With STEMI

**DOI:** 10.1016/j.jaccas.2025.105668

**Published:** 2025-10-07

**Authors:** Katherine A. Burns, Saeid Mirzai, Zachary D. Pruitt, Katherine Mayle, Luke Peters, Manrique Alvarez, Hassan A. Khan, P. Matthew Belford, Graham V. Byrum

**Affiliations:** Department of Cardiovascular Medicine, Wake Forest University School of Medicine, Winston-Salem, North Carolina, USA

**Keywords:** mortality prediction, out-of-hospital cardiac arrest, percutaneous coronary intervention, risk stratification, ST-elevation myocardial infarction

## Abstract

**Background:**

Out-of-hospital cardiac arrest (OHCA) with ST-segment elevation myocardial infarction (STEMI) carries a high mortality risk. The MIRACLE2 and Cardiac Arrest Hospital Prognosis (CAHP) scores show promise for neurological outcome prediction, but their performance for in-hospital mortality in patients with OHCA-STEMI remains unclear.

**Project Rationale:**

Accurate early mortality prediction could guide decisions regarding percutaneous coronary intervention and resource allocation in high-risk patients with OHCA-STEMI.

**Project Summary:**

We analyzed 120 patients with OHCA-STEMI (2019-2023), comparing MIRACLE2 and CAHP for mortality prediction. Both demonstrated excellent discrimination (MIRACLE2 0.855 vs CAHP AUC 0.878, *P* = 0.360). Missing data impacted performance, with CAHP more vulnerable than MIRACLE2. In combined modeling, CAHP retained independent significance while MIRACLE2 did not. Traditional cutoffs (MIRACLE2 ≥5, CAHP >200) had high specificity but poor sensitivity (<25%). Patients with either or both scores elevated by traditional cutoffs demonstrated >90% mortality. Derived cutoffs (MIRACLE2 ≥4, CAHP ≥158) improved sensitivity (MIRACLE2 75.9%, CAHP 81.0%).

**Take-Home Messages:**

MIRACLE2 and CAHP are strong predictors of mortality in OHCA-STEMI, and using both in parallel improves identification of patients unlikely to benefit from percutaneous coronary intervention.

## Graphical Abstract

**Figure:**
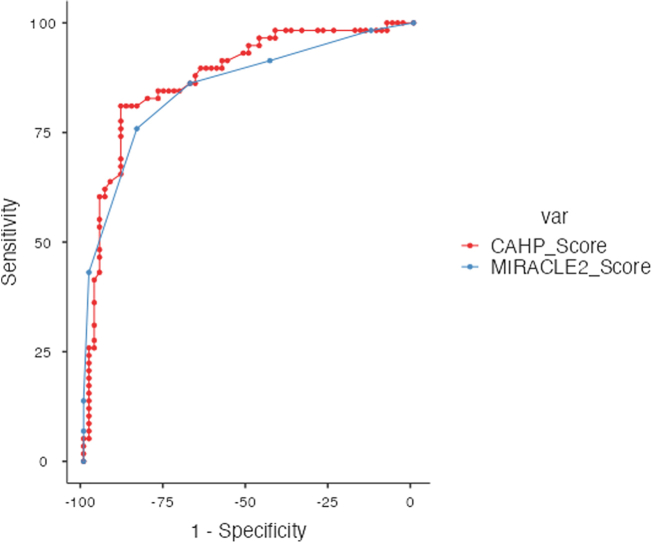

Out-of-hospital cardiac arrest (OHCA) complicated by ST-segment elevation myocardial infarction (STEMI) represents a critical intersection of 2 life-threatening conditions, with in-hospital mortality rates of almost 40%.^1^ While emergency percutaneous coronary intervention (PCI) remains the standard of care, the substantial resource requirements, procedural risks, and opportunity costs associated with emergent cardiac catheterization necessitate accurate risk-stratification tools to optimize patient selection and avoid futile interventions that expose patients to unnecessary harm while consuming limited healthcare resources.

**Take-Home Messages**

• MIRACLE2 is practical and robust, while cardiac arrest hospital prognosis adds complementary prognostic value.
• Patients with either or both scores high by traditional cutoffs had mortality exceeding 90%, supporting their combined use to identify those unlikely to benefit from percutaneous coronary intervention.
• Exploratory mortality-specific cutoffs showed promise but require external validation, and larger cohorts will be needed to guide development of an optimized out-of-hospital cardiac arrest with ST-segment elevation myocardial infarction score.

The MIRACLE2 and Cardiac Arrest Hospital Prognosis (CAHP) scores have demonstrated utility in predicting neurological outcomes after OHCA.^2^, ^3^, ^4^, ^5^ However, their role in predicting mortality specifically in OHCA-STEMI remains uncertain, and whether the combined application improves risk stratification is unknown. This represents an important gap in optimizing care for these high-acuity patients requiring immediate, resource-intensive interventions.

## Case Summary Prompting Project Launch

A 61-year-old male presented after witnessed OHCA with initial ventricular fibrillation. After achieving return of spontaneous circulation, electrocardiography revealed anterior STEMI. His early postarrest neurologic status was indeterminate. His calculated MIRACLE2 score was 2, suggesting low risk, while his CAHP score was 210, indicating high risk for poor neurologic outcome. Following our standard MIRACLE2-predominant approach, the patient underwent emergent PCI with successful revascularization.

Postprocedurally, the patient had an initially stable course but subsequently developed progressive cardiogenic shock over the following days, requiring mechanical circulatory support consideration. However, his neurologic examination remained equivocal, making neurologic prognostication challenging. After multidisciplinary consultation, the family ultimately declined mechanical circulatory support, given the uncertain neurologic prognosis, and opted for comfort care. The patient died on hospital day 3. This case highlighted how discordant risk scores complicate prognostic ability and prompted our systematic evaluation of a multirisk score assessment strategy in our OHCA-STEMI population.

## Project Rationale

Our current institutional protocols for patients with OHCA-STEMI primarily rely on the MIRACLE2 score with traditional cutoffs to guide aggressive intervention decisions. However, we observed that many patients categorized as low or medium risk by MIRACLE2 still died, suggesting under-recognition of mortality risk. The CAHP score, which is more data-intensive, may capture additional prognostic factors not reflected in MIRACLE2. Understanding the comparative performance of these scores, their optimal use in combination, and the potential for mortality-specific cutoffs could enhance patient selection for invasive management while reducing exposure to futile care.

## Project Description

We conducted a retrospective analysis using the American College of Cardiology National Cardiovascular Data Registry at our institution from 2019 to 2023. Adult patients aged 18 years and older with OHCA who underwent emergent cardiac catheterization for STEMI were included. Patient demographics, arrest characteristics, interventional details, and clinical outcomes were obtained from the registry, which uses uniform definitions, standardized data elements, and rigorous quality-control processes as established by the American College of Cardiology National Cardiovascular Data Registry.^6^

Both MIRACLE2^3^ and CAHP^4^ scores were calculated using available registry data. For missing variables, standardized assumptions were applied, including pH 7.4 if unknown, 5-minute intervals for unknown collapse-to-cardiopulmonary resuscitation and cardiopulmonary resuscitation to return of spontaneous circulation durations, reactive pupils if unknown for MIRACLE2, and no epinephrine if unknown and ≥3 if epinephrine drip for CAHP. The primary outcome was in-hospital mortality.

Statistical analysis included receiver operating characteristic curve analysis with area under the curve comparison using DeLong test, optimal cutoff determination using Youden index, and multivariable logistic regression with stepwise selection. Combined score performance was assessed using four-group risk stratification. Confidence intervals (CI) for area under the curve estimates were derived using 2,500 bootstrap replicates. Categorical variables were presented as frequency and percentage, and continuous variables as mean ± standard deviation or median with interquartile range. Odds ratios (OR) with 95% CI were calculated for mortality associations. Statistical significance was set at *P* < 0.05. Statistical analyses were conducted in R version 4.3.2 and JMP Pro 17.

## Project Deliverables

**Figure 1:**
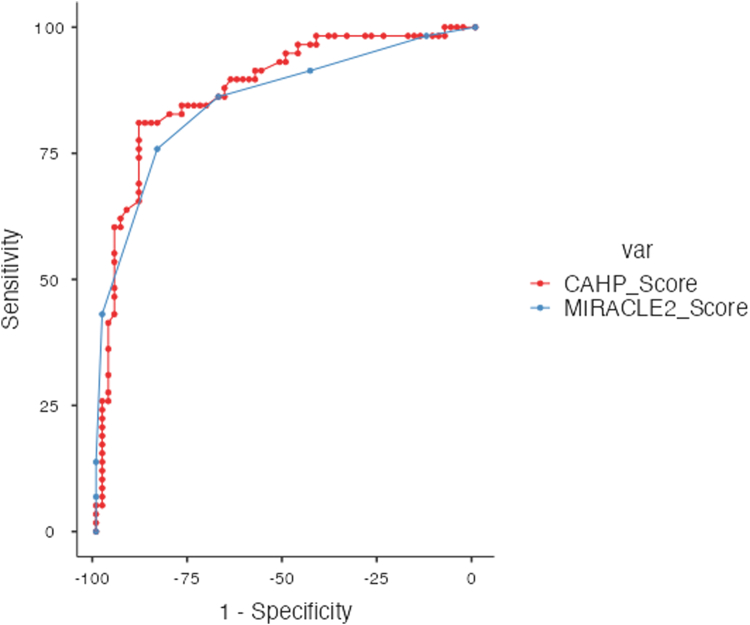
Receiver Operating Characteristic (ROC) Curves for the MIRACLE2 Score (Blue Line) and CAHP Score (Red Line) for Predicting In-Hospital Mortality in Out-of-Hospital Cardiac Arrest With ST-Elevation Myocardial Infarction

Our analysis of 120 patients with OHCA-STEMI revealed a mean age of 61.1 years, with 75% male predominance and 48.3% in-hospital mortality (Table 1). Both scoring systems demonstrated excellent discriminatory ability with overlapping CI. MIRACLE2 achieved an area under the curve of 0.855 (95% CI: 0.774-0.909) compared to CAHP at 0.878 (95% CI: 0.799-0.930), with no statistically significant difference (*P* = 0.360) (Figure 1).

**Table 1:** Baseline Characteristics of the Study Population (N = 120)

Demographics	
Age, years	61.1 ± 11.4
Female sex	30 (25.0%)
Race	
White	99 (82.5%)
Black	16 (13.3%)
Other	5 (4.2%)
Insurance status	
Private	46 (38.3%)
Public	47 (39.2%)
Uninsured	27 (22.5%)
Clinical characteristics	
MIRACLE2 score	3 (1-4)
CAHP score	151 (110-176)
Comorbidities	
Cerebrovascular disease	13 (10.8%)
Peripheral arterial disease	4 (3.3%)
Chronic lung disease	16 (13.3%)
Prior PCI	16 (13.3%)
Diabetes	23 (19.2%)
Cardiac status	
Ejection fraction (%) (missing in n = 30)	52.6 ± 11.2
Cardiogenic shock	40 (33.3%)
Multivessel disease	63 (52.5%)
Chronic total occlusion	3 (2.5%)
In-stent thrombosis	1 (0.8%)
Clinical outcomes	
In-hospital mortality	58 (48.3%)
Discharge to hospice (missing in n = 57)	2 (3.2%)
Length of stay, days (for n = 62 without in-hospital mortality)	5.0 (3.2-9.0)

Values are n (%), mean ± SD, or median (IQR).

CAHP = Cardiac Arrest Hospital Prognosis; PCI = percutaneous coronary intervention.

Missing data substantially impacted score calculation feasibility, affecting 45.5% of patients for MIRACLE2 and 67.5% for CAHP (Table 2). When complete data were available, MIRACLE2 remained relatively stable (AUC 0.841 vs 0.868) while CAHP performance improved (AUC 0.942 vs 0.851), suggesting MIRACLE2's superior resistance to missing data.

**Table 2:** Impact of Missing Data on MIRACLE2 and CAHP Predictive Performance for In-Hospital Mortality

Score	Patients With Missing Data, n/N (%)	Complete Data		Missing Data	
		AUC	Unadjusted OR (95% CI, *P* value)	AUC	Unadjusted OR (95% CI, *P* value)
MIRACLE2 score	35/77 (45.5)	0.841	5.28 (1.92-14.53, ***P* = 0.001**) per 1 SD increase	0.868	6.38 (2.15-18.89, ***P* < 0.001**) per 1 SD increase
CAHP score	52/77 (67.5)	0.942	26.23 (2.19-313.44, ***P* = 0.010**) per 1 SD increase	0.851	4.51 (2.02-10.11, ***P* < 0.001**) per 1 SD increase
Combined scores^a^	56/77 (72.7)	0.981	-	0.861	-

Missingness data were unavailable in 43 patients. AUC calculated using raw variables; ORs calculated using z-score normalized variables.

AUC = area under the receiver operating characteristic curve; CAHP = Cardiac Arrest Hospital Prognosis; CI = confidence interval; OR = odds ratio.

a Model including both MIRACLE2 and CAHP scores as continuous variables.

Traditional cutoffs demonstrated high specificity exceeding 95% but poor sensitivity below 25% for both scoring systems. Derived optimal cutoffs using Youden index improved sensitivity substantially while maintaining good specificity (Table 3). For MIRACLE2, the derived cutoff (≥4) achieved 75.9% sensitivity and 83.9% specificity compared to the traditional cutoff (≥5) performance of 43.1% sensitivity and 98.4% specificity. Similarly, CAHP's derived cutoff (≥158) achieved 81.0% sensitivity and 88.7% specificity compared to the traditional cutoff (>200) performance of 22.4% sensitivity and 98.4% specificity.

**Table 3:** Test Performance of MIRACLE2 and CAHP Cutoffs for In-Hospital Mortality

Score and Cutoff	Patients Above Cutoff, n/N (%)	Sensitivity, %	Specificity, %	PPV, %	NPV, %
MIRACLE2 Score					
Traditional cutoff (≥5)	26/120 (21.7)	43.1	98.4	96.2	64.9
Derived cutoff (≥4)	54/120 (45.0)	75.9	83.9	81.5	78.8
CAHP score					
Traditional cutoff (>200)	14/120 (11.7)	22.4	98.4	92.9	57.6
Derived cutoff (≥158)	54/120 (45.0)	81.0	88.7	87.0	83.3
Combined scores					
Both high (traditional)^a^	11/120 (9.2)	17.2	98.4	90.9	56.0
Both high (derived)^b^	46/120 (38.3)	72.4	93.5	91.3	78.4

CAHP = Cardiac Arrest Hospital Prognosis; NPV = negative predictive value; PPV = positive predictive value.

a Traditional cutoffs: MIRACLE2 ≥5 and CAHP >200.

b Derived cutoffs: MIRACLE2 ≥4 and CAHP ≥158.

Multivariable analysis revealed that when both scores were included simultaneously, MIRACLE2 did not reach statistical significance (adjusted OR: 2.20, 95% CI: 0.94-5.14, *P* = 0.069) while CAHP retained independent predictive significance (adjusted OR: 3.84, 95% CI: 1.53-9.68, *P* = 0.004) as demonstrated in Table 4. Cardiogenic shock also remained an independent predictor (adjusted OR: 3.85, 95% CI: 1.17-12.73, *P* = 0.027).

**Table 4:** Logistic Regression Analysis of Continuous (Z-Score Normalized) MIRACLE2 and CAHP Scores for In-Hospital Mortality

	Unadjusted OR (95% CI), *P* value	Adjusted OR (95% CI), *P* value
MIRACLE2 score (per 1 SD increase)	5.94 (3.20-10.99), **<0.001**	2.20 (0.94-5.14), 0.069
CAHP score (per 1 SD increase)	7.33 (3.69-14.57), **<0.001**	3.84 (1.53-9.68), **0.004**
Demographics		
Age (per 10-year increase)	1.01 (0.98-1.05), 0.385	-
Male sex	0.91 (0.40-2.09), 0.833	-
White race	0.82 (0.32-2.11), 0.683	-
Uninsured status	0.45 (0.18-1.10), 0.080	-
Comorbidities		
Cerebrovascular disease	1.82 (0.56-5.94), 0.318	-
Peripheral arterial disease	0.34 (0.03-3.41), 0.363	-
Chronic lung disease	2.67 (0.86-8.22), 0.087	-
Prior PCI	1.94 (0.66-5.74), 0.229	-
Diabetes	2.99 (1.13-7.93), **0.027**	3.47 (0.66-18.17), 0.141
Cardiac status		
Ejection fraction (per 10-fold increase)	0.06 (0.00-2.59), 0.146	-
Cardiogenic shock	6.76 (2.82-16.22), **<0.001**	3.85 (1.17-12.73), **0.027**
Multivessel disease	1.23 (0.60-2.52), 0.571	-
Treatment		
Procedure time (per 10-fold increase)	13.49 (2.51-72.53), **0.002**	7.10 (0.72-69.89), 0.093

Variables with *P* < 0.05 in univariable analysis were included in the multivariable model.

CAHP = Cardiac Arrest Hospital Prognosis; CI = confidence interval; OR = odds ratio; PCI = percutaneous coronary intervention.

Four-group risk stratification using both scores revealed distinct mortality patterns as detailed in Table 5. Using traditional cutoffs, patients with neither score elevated had 33.0% mortality, those with MIRACLE2 elevation alone had 100% mortality, CAHP elevation alone had 100% mortality, and both scores elevated had 90.9% mortality. Using derived cutoffs, patients with neither score elevated had 15.5% mortality, those with MIRACLE2 elevation alone had 25.0% mortality, CAHP elevation alone had 62.5% mortality, and both scores elevated had 91.3% mortality. Consistent with the improved sensitivity observed for individual scores, the derived cutoffs provided superior discriminative ability compared to traditional thresholds and individual score utilization, although these cutoffs require external validation, having been derived from the same cohort.

**Table 5:** Logistic Regression Analysis of MIRACLE2 and CAHP Risk Score Groups for In-Hospital Mortality

Risk Score Category	Mortality, n/N (%)	Unadjusted OR (95% CI), *P* value	Adjusted OR (95% CI), *P* value^a^
MIRACLE2 + CAHP Scores (Traditional)			
Neither high-risk (Group 0)	30/91 (32.97)	Reference	Reference
MIRACLE2 high-risk only (Group 1)	15/15 (100.0)	Not estimable^b^	Not estimable^b^
CAHP high-risk only (Group 2)	3/3 (100.0)	Not estimable^b^	Not estimable^b^
Both high-risk (Group 3)	10/11 (90.91)	20.33 (2.49-166.31), **0.005**	15.32 (1.52-154.61), **0.021**
MIRACLE2 + CAHP scores (derived)			
Neither high-risk (Group 0)	9/58 (15.5)	Reference	Reference
MIRACLE2 high-risk only (Group 1)	2/8 (25.0)	1.81 (0.32-10.45), 0.505	1.81 (0.25-12.88), 0.555
CAHP high-risk only (Group 2)	5/8 (62.5)	9.07 (1.84-44.86), **0.007**	7.91 (1.30-48.14), **0.025**
Both high-risk (Group 3)	42/46 (91.3)	57.17 (16.41-199.10), **<0.001**	55.32 (13.96-219.30), **<0.001**

High-risk cutoffs: traditional = MIRACLE2 ≥5 and CAHP >200; derived = MIRACLE2 ≥4 and CAHP ≥158.

CAHP = Cardiac Arrest Hospital Prognosis; CI = confidence interval; OR = odds ratio.

a Adjusted for diabetes, cardiogenic shock, and procedure time (log-transformed [base 10]).

b Odds ratio not estimable due to 100% mortality rate in the group.

## Project Outcome, Impact, and Future Directions

Our analysis demonstrates that both MIRACLE2 and CAHP are strong predictors of mortality in patients with OHCA-STEMI, with CAHP showing some superior prognostic performance. Importantly, patients classified as high risk by either score using traditional cutoffs had mortality rates exceeding 90%, highlighting that reliance on MIRACLE2 alone may fail to identify some patients at very high risk of death.

These findings have prompted institutional discussions about using both scores together to guide decisions regarding PCI candidacy. Incorporating CAHP alongside MIRACLE2 may help avoid exposing patients to futile interventions, support more informed conversations with families about prognosis, and reduce the high costs and resource utilization associated with procedures unlikely to provide benefit in patients who are critically ill post arrest.

In exploratory analyses, derived cutoffs improved sensitivity while maintaining good specificity, identifying more patients with very high mortality risk and further enhancing prognostic ability. However, these thresholds were generated in the same cohort and require external validation before clinical adoption. Future directions include validation of derived cutoffs in larger populations and development of a novel, institution-specific OHCA-STEMI score incorporating the most predictive variables to optimize mortality prediction.

## Discussion

Our study demonstrates that both MIRACLE2 and CAHP scores provide excellent prognostic information for patients with OHCA-STEMI, and that using both scores together enhances mortality prediction beyond either score alone. The independent significance of CAHP, while MIRACLE2 lost statistical significance in the combined model, suggests CAHP may provide superior prognostic information for mortality prediction in patients with OHCA-STEMI. Consistent with this, patients with either or both scores elevated by traditional cutoffs had mortality exceeding 90%, suggesting these individuals may be candidates for conservative management rather than aggressive invasive interventions.

These findings are directly relevant for clinical decision-making. MIRACLE2, the tool currently used at our institution, demonstrated robustness despite missing data, aligning with the original MIRACLE2 development objectives of creating a more practical scoring system.^3^ Importantly, our results highlight that adding CAHP improves prognostication, particularly when complete data are available, where its performance is strengthened. Using both scores in parallel may better identify patients who are at too high risk to benefit from PCI, avoid procedures that carry risks of intraprocedural deterioration, and support more transparent counseling with families regarding goals of care.

Our exploratory analyses suggest that mortality-specific cutoffs may improve sensitivity while retaining specificity, identifying a larger proportion of patients with a very poor prognosis. While these findings are hypothesis-generating and require validation in larger, external cohorts, they provide a framework for refining prognostic assessment in OHCA-STEMI. Ultimately, our goal is to confirm these results in multicenter populations and to develop a novel risk score incorporating the most predictive elements of existing tools.

Limitations of our analysis include a single-institution design, relatively small sample size, retrospective methodology, and reliance on assumptions about missing data that may not reflect true clinical scenarios. The derivation cohort approach limits immediate generalizability. In addition, our analysis focused on in-hospital mortality rather than longer-term functional outcomes that may be equally relevant for intervention decisions.

## Conclusions

In patients with OHCA-STEMI, both MIRACLE2 and CAHP demonstrated excellent prognostic performance for in-hospital mortality. While MIRACLE2 remains practical and robust despite missing data, CAHP adds complementary predictive value, and together, the scores identified patients with mortality exceeding 90% when either or both were high risk by traditional cutoffs. These findings support consideration of dual scoring in clinical practice to recognize patients unlikely to benefit from PCI, inform discussions with families, and reduce exposure to futile care.

### Data Availability

Data are available upon reasonable request to the corresponding author, subject to institutional review board approval and data sharing agreements.

## Funding Support and Author Disclosures

The authors have reported that they have no relationships relevant to the contents of this paper to disclose.
